# Angiomirs expression profiling in diffuse large B-Cell lymphoma

**DOI:** 10.18632/oncotarget.6624

**Published:** 2015-12-15

**Authors:** Natália M. Borges, Marcela do Vale Elias, Veruska L. Fook-Alves, Tathiana A. Andrade, Marina Lourenço de Conti, Mariana Petaccia Macedo, Maria Dirlei Begnami, Antônio Hugo J. F. M. Campos, Leina Yukari Etto, Adriana Bruscato Bortoluzzo, Antonio C. Alves, Ken H. Young, Gisele W. B. Colleoni

**Affiliations:** ^1^ Departamento de Oncologia Clínica e Experimental, Universidade Federal de São Paulo, São Paulo, Brazil; ^2^ A.C. Camargo Cancer Center, São Paulo, Brazil; ^3^ Insper Institute of Education and Research, São Paulo, Brazil; ^4^ Departamento de Patologia, Universidade Federal de São Paulo, São Paulo, Brazil; ^5^ Department of Hematopathology, MD Anderson Cancer Center, Houston, Texas, USA

**Keywords:** lymphoma, angiogenesis, microRNAs

## Abstract

Despite advances in treatment, 30% of diffuse large B-cell lymphoma (DLBCL) cases are refractory or relapse after chemoimmunotherapy. Currently, the relationship between angiogenesis and angiomiRs in DLBCL is unknown. We classified 84 DLBCL cases according to stromal signatures and evaluated the expression of pro- and antiangiomiRs in paraffin embedded tissues of DLBCL and correlated them with microvascular density (MVD). 40% of cases were classified as stromal-1, 50% as stromal-2 and 10% were not classified. We observed increased expression of proangiomiRs Let-7f, miR-17, miR-18a, miR-19b, miR-126, miR-130a, miR-210, miR-296 and miR-378 in 14%, 57%, 30%, 45%, 12%, 12%, 56%, 58% and 48% of the cases, respectively. Among antiangiomiRs we found decreased expression of miR-16, miR-20b, miR-92a, miR-221 and miR-328 in, respectively, 27%, 71%, 2%, 44% and 11%. We found association between increased expression of proangiomiRs miR-126 and miR-130a and antiangiomiR miR-328 and the subtype non-GCB. We found higher levels of the antiangiomiRs miR-16, miR-221 and miR-328 in patients with low MVD and stromal-1 signature. IPI and CD34 confirmed independent impact on survival of the study group. None of the above angiomiRs showed significance as biomarker in an independent serum samples cohort of patients and controls. In conclusion, we confirmed association between antiangiomiRs miR-16, miR-221 and miR-328 and stromal-1 signature. Four angiomiRs emerged as potential therapeutic targets: proangiomiRs miR-17, miR-210 and miR-296 and antiangiomiR miR-20b. Although the four microRNAs seem to be important in DLBCL pathogenesis, they were not predictive of DLBCL onset or relapse in the serum independent cohort.

## INTRODUCTION

Angiogenesis is the production of new capillaries from a pre-existing vascular bed. It occurs physiologically during embryonic development, wound healing and in the female reproductive tract during the menstrual cycle [1, 2]. It is important in the proliferation and development of metastasis in solid malignancies, since without the formation of vessels to support the tumor, it does not grow more than 1 or 2 mm [3, 4]. Thus, inhibition of angiogenesis can suppress the growth and the potential of tumor invasion and it could be an important strategy in controlling malignancies [1, 5]. The study of tumor angiogenesis in hematologic malignancies, like non-Hodgkin's lymphomas (NHL), is a relevant issue to be explored, bringing new expectations to the therapeutic, and mostly, to the evolution of the patients who suffer from this prevalent neoplasia.

MicroRNAs (miRNAs) are a class of highly conserved non-coding RNAs, with around 20-22 nucleotides. They have been described as regulators of encoding protein genes and some of them have a role in the control of many aspects of angiogenesis [6, 7]. The identification of these miRNAs has opened new paths in the therapeutic of vascular and oncologic diseases, since they are candidates to target specific therapies [6, 7]. In this study we extended one former endeavor [8, 9] and evaluated the expression of angiogenesis-related miRNAs in paraffin embedded tissues of diffuse large B-cell lymphomas (DLBCL). DLBCL constitute the majority of the diagnosed patients with B-NHL and it can be used as a model to explore the angiogenesis as a therapeutic target for other types of lymphomas. Besides, we intended to explore the importance of the tumor microenvironment and to classify DLBCL cases according to the prognostic signatures described in 2008 by Lenz *et al.* [10], i.e., stromal-1 and stromal-2. The stromal-1 signature reflects the extracellular matrix deposition, while the stromal-2 signature represents tumors with higher MVD.

Currently, DLBCL cases show favorable response to standard immunochemotherapy (R-CHOP - rituximab, cyclophosphamide, doxorubicin, vincristine and prednisone). However, 10%-15% of the patients will be primarily refractory to this treatment and around 20%-25% will relapse after the initial response [11, 12]. Therefore, one third of the DLBCL cases who do not respond to the standard therapy need a different approach. The combined analysis of the angiogenesis process in DLBCL using the expression of the proteins related to the stromal signature and the expression of miRNAs could estimate the importance of these miRNAs expression in the pathogenesis and the treatment of DLBCL patients. The result of this novel approach may suggest new therapeutic strategies against DLBCL, throughout the development of miRNAs inhibitors of proangiomiRs or miRNAs mimics of antiangiomiRs.

## RESULTS

The 84 DLBCL cases were classified according to the algorithm of Hans *et al.* [13]. 46.4% of the cases were identified as GCB, 36.9% as ABC (non-GCB) and 16.7% were unclassifiable. In the automated analysis of MVD, the results of CD34 expression were divided into quartiles according to the median number of CD34+ objects per 100 μm^2^ TMA area, as shown below: Quartile I 24.93 to 738.41; Quartile II 741.17 to 2522.48; Quartile III 2549.68 to 5687.33; Quartile IV 6743.04 to 25424.57. In the manual analysis of MVD, the results of CD34 expression were also divided in quartiles, according to the median number of blood vessels by TMA area, as shown below: Quartile I 12-71; Quartile II 71-104; Quartile III 105-136; Quartile IV 137-297. We found positive correlation (Pearson correlation coefficient, p=0.0163) between the assessment of MVD by the two methods. (Supplementary Figure 1). There was no statistically significant difference among the clinical variables gender, age, stage, IPI and molecular subtypes (Table 2) and according to their distribution in the groups with low MVD (quartiles I/II) and high MVD (quartiles III/IV) using the data obtained from the automated or the manual counting of MVD. Among the 84 patients, 13 (15.5%) were treated with R-CHOP. The others received antracyclin-based regimen without rituximab. The small number of patients treated with R-CHOP is justified by the introduction of this standard therapy in the Brazilian Health System only in 2007 (most of our patients were diagnosed before this year).

**Figure 1:**
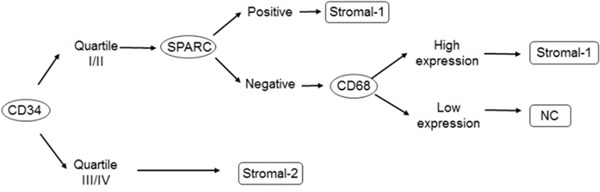
Algorithm proposal for stromal signature classification, by immunohistochemistry Abbreviations: SPARC = secreted protein, acidic and rich in cystein; stromal-1 and stromal-2 = stromal signatures; NC = not classified.

Using the algorithm shown in Figure 1, 40% of the cases were classified as stromal-1, 50% as stromal-2 and 10% were not classified. We could not find associations between stromal signatures and clinical variables.

**Figure 2:**
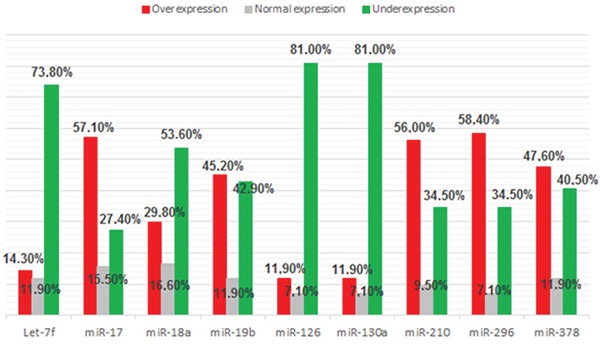
Frequency of proangiomiRs expression (Let-7f, miR-17, miR-18a, miR-19b, miR-126, miR-130a, miR-210, miR-296 and miR-378) in 84 samples of DLBCL

**Figure 3:**
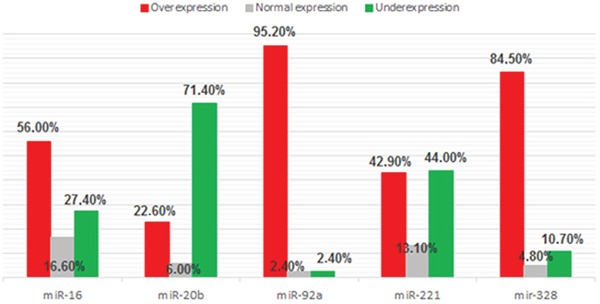
Frequency of antiangiomiRs expression (miR-16, miR-20b, miR-92a, miR-221 and miR-328) in 84 samples of DLBCL

We observed increased expression of proangiomiRs Let-7f, miR-17, miR-18a, miR-19b, miR-126, miR-130a, miR-210, miR-296 and miR-378 in 14%, 57%, 30%, 45%, 12%, 12%, 56%, 58% and 48% of the cases, respectively. Among antiangiomiRs we found decreased expression of miR-16, miR-20b, miR-92a, miR-221 and miR-328 in 27%, 71%, 2%, 44% and 11% of the cases, respectively (Figures 2 and 3).

**Figure 4:**
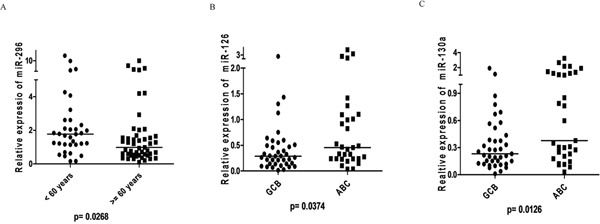
ProangiomiRs **A.** Expression of miR-296 according to age at DLBCL diagnosis showed higher expression in patients aged <60 years (Mann-Whitney, p=0.0268); **B.** miR-126 expression according to molecular subtypes of Hans algorithm showing higher expression in ABC subtype (Mann-Whitney, p=0.0374); **C.** miR-130a expression according to molecular subtypes of Hans algorithm showing higher expression in ABC subtype (Mann-Whitney, p=0.0126).

**Figure 5:**
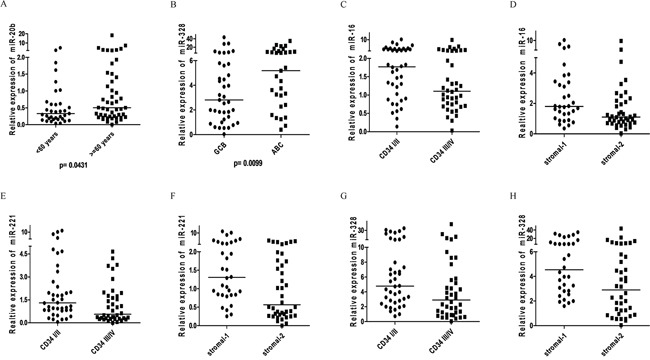
AntiangiomiRs **A.** Expression of miR-20b according to age at DLBCL diagnosis showed higher expression in patients aged >= 60 years (Mann-Whitney, p=0.0431); **B.** miR-328 expression according to molecular subtypes of Hans algorithm showing higher expression in ABC subtype (Mann-Whitney, p=0.0099); **C.** miR-16 expression in DLBCL according to CD34 (MVD - Manual counting) (p=0.0331, Mann-Whitney). The expression of CD34 (MVD) is ranked quartiles: I and II considered as low expression; III and IV considered as high expression. Number of cases examined = 80/84 (4 cases not available for CD34 expression); **D.** miR-16 expression in DLBCL according to stromal signature (p=0.0146, Mann-Whitney); **E.** miR-221 expression in DLBCL according to CD34 (MVD - manual counting) (p=0.0182, Mann-Whitney). The expression of CD34 (MVD) is ranked quartiles: I and II considered as low expression; III and IV considered as high expression. Number of cases analyzed = 80/84 (4 cases not available to CD34 expression); **F.** miR-221 expression in DLBCL according to stromal signature (p=0.0076, Mann-Whitney). **G.** Expression of miR-328 in DLBCL according to CD34 (MVD - manual counting) (p=0.0129, Mann Whitney). The expression of CD34 (MVD) is ranked quartiles: I and II considered as low expression; III and IV considered as high expression. Number of cases examined = 80/84 (4 cases not amenable for CD34 expression); **H.** miR-328 expression in DLBCL according to stromal signature (p=0.0099, Mann-Whitney).

We analyzed the correlation between the 14 angiomiRs and clinical variables, molecular subtypes, MVD (automated and manual counting) and stromal signatures. We found association between the expression of two angiomiRs and the clinical variable age (< 60 years *vs* ≥ 60 years); increased expression of miR-296 and miR-20b was associated with age < 60 years and age ≥ 60 years, respectively (Figures 4 and 5). No associations were found between the angiomiRs expression and the other clinical variables gender, staging and IPI.

Regarding the molecular subtypes, we found association between increased expression of three angiomiRs (miR-126, miR-130a and miR-328) and the subtype ABC (Figures 4 and 5).

We did not find statistically significant differences between the relative expression of the angiomiRs and the MVD evaluated by automated counting. However, we found expression values of the angiomiRs miR-16, miR-221 and miR-328 compatible with the MVD evaluated by manual counting, i.e., higher levels of the three antiangiomiRs in the setting of cases classified as low MVD (quartiles I/II) (Figure 5). The Supplementary Figure 2 shows the expression level of angiomiRs (increased expression, decreased expression and normal expression), according to stromal-1 and stromal-2 signatures.

We found statistically significant differences between the relative expression of angiomiRs Let-7f, miR-126, miR-210, miR-378, miR-16, miR-221 and miR-328 and the stromal-1 and stromal-2 signatures. However, only for miR-16, miR-221 and miR-328 we verified values of angiomiR expression compatible with the stromal signature, i.e., higher levels of antiangiomiRs on stromal-1 signature (Figure 5).

**Figure 6:**
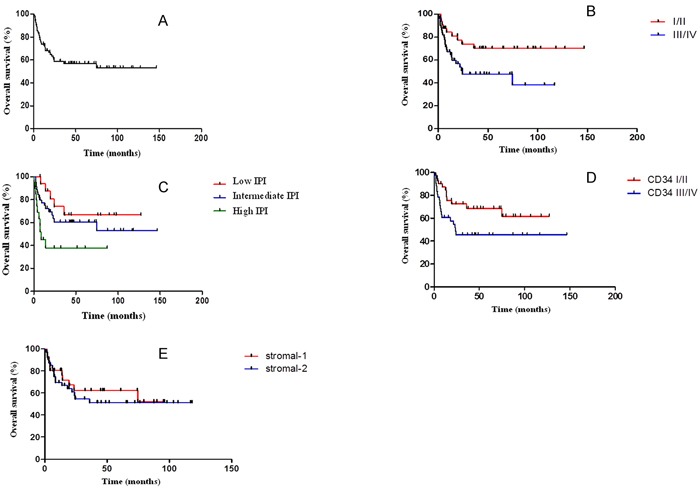
**A.** Overall survival curve of the 84 patients included in the study. The median overall survival has not been reached, with a maximum follow-up of 146.53 months; **B.** Overall survival curve according to Ann Arbor Staging. The median overall survival in clinical stage I/II group has not been reached, while in the III/IV group was 24.27 months (log-rank, p=0.0256); **C.** Overall survival curve according to the International Prognostic Index (IPI). The median overall survival in the low and intermediate IPI groups was not achieved and was 8.47 months in the high IPI group (log-rank, p=0.0236; IPI 0-1: low; IPI 2-3: intermediate; IPI 4-5: high); **D.** Overall survival curve according to MVD (automated counting (CD34+/100μm^2^). The median overall survival in groups of quartiles I/II and quartiles III/IV has not been reached (log-rank, p=0.0363); **E.** Overall survival curve of the patients according to stromal signature. The median overall survival in the stromal-1 and stromal-2 groups was not reached (log-rank, p=0.6157).

The median overall survival of the 84 patients was not reached, with a maximum follow-up of 146.53 months. This fact has probably been due to the loss of follow-up of 28 patients (33.3%). Of these, six patients (7.14%) were discharged after 5 years and returned to their hometown for follow-up. By the end of the study, 33 patients (39.3%) had died (survival from 0.63 to 74.67 months) and 23 patients (27.4%) remained alive and receiving outpatient treatment (survival from 19.27 to 146.53 months). We found statistically significant differences when comparing Ann Arbor I/II with Ann Arbor III/IV stages (Log-rank test, p=0.0256). We also found a statistically significant difference when comparing the groups classified as low, intermediate and high International Prognostic Index (Log-rank, p=0.0236). Another statistically significant difference was observed when comparing the MVD groups classified as quartiles I/II *versus* quartiles III/IV according to the automated counting (CD34+/100μm^2^). (Table 3) No relevant difference in survival according to the stromal classification or clinical parameters was found (Figure 6).

We conducted multivariate analysis using the Cox regression model including variables such as staging, IPI and CD34 (automated counting), selected as important in survival in univariate analysis. IPI and CD34 confirmed independent impact on survival of the study group (p=0.044 and 0.048, respectively). Patients with high-risk IPI showed likelihood of evolving to death 3.4 times higher than the rest of the group (95% CI 1.168 to 9.938), while patients with higher MVD (quartiles III and IV) showed likelihood of evolving to death 2.0 times higher than the rest of the group (95% CI 1.008 to 4.193).

The angiomiRs identified as differentially expressed in DLBCL tissue (proangiomiRs miR-17, miR-210, miR-296 and antiangiomiR miR-20b) were evaluated in an independent cohort of serum samples from patients with DLBCL at diagnosis and at the time of relapse. After evaluation of RNU44 (threshold cycle < 38), 6/10 (60%) controls, 21/39 (53.8%) DLBCL cases at diagnosis and 7/19 (52.6%) relapsed DLBCL cases were considered valid for the miRNA expression evaluation. Although the four microRNAs seem to be important in DLBCL pathogenesis, no present difference was observed when the three groups of patients were analyzed (Supplementary Figure 3).

## DISCUSSION

In the present study, we classified DLBCL according to stromal-1 and stromal-2 signatures and evaluated the expression of miRNAs involved in angiogenesis, divided into proangiomiRs and antiangiomiRs. We observed correlation between the expression of stromal signatures and angiomiRs. Differentially expressed miRNAs can offer new possibilities of therapeutic interventions, especially considering that the same miRNA can potentially interfere in multiple oncogenic pathways [14, 15]. Besides, since angiogenesis is an important feature involved in the development and maintenance of malignant neoplasias, the data identified herein may be applicable to other types of cancer [16].

46.4% of the 84 DLBCL cases were classified as GCB subtype and 36.9% as ABC (non-GCB), similar to the report by Hans *et al.* [13]. Concerning the expression of CD34, we used two methods to analyze the MVD expression: automated and manual counting. For better classification, the results of CD34 expression with either the manual or the automated method were divided into quartiles, as done in other studies [17, 18]. We found a positive correlation between both methods and we stress that this is the first study that compares the evaluation of MVD by the two systems in DLBCL. We found no statistically significant difference between clinical variables and molecular subtypes, according to the patients' distribution in low MVD (quartiles I/II) and high MVD (quartiles III/IV) neither in automated nor manual counting of MVD.

The stromal signatures were determined from gene expression and subsequently correlated with immunohistochemical markers [10]. In the present study, we chose SPARC (secreted protein, acidic and rich in cysteine) and CD68 as stromal-1 markers. Seeking the best alternative to classify cases according to the stromal signature, we made a third categorization using CD34, followed by SPARC and CD68 sequentially (Figure 1). We classified 40% as stromal-1, 50% as stromal-2 and 10% as unclassified. Such effort minimized the percentage of cases that could not be classified and would not participate in further evaluations, including associations with angiomiRs. Although this sequential approach have not been described previously, it seems logical because the MVD manual counting correlated well with the automated counting, and SPARC and CD68 seem to identify the same population of cells. Another reason to use CD34 as a starting point for the classification is our focus on epigenetic mechanisms of angiogenesis control. This way, we sought associations between the expression of the 14 angiomiRs and clinical features, molecular subtypes, MVD and stromal signatures.

We also found increased expression of proangiomiR miR-130a in non-GCB patients. In the non-GCB DLBCL there is constitutional activation of the NF-kB pathway [19, 20]. Zhang *et al.* [21] observed that the increased expression of NF-kB or miR-130a promoted tumor growth. They described that TNF-α, NF-κB and miR-130a operate in negative feedback: lower TNF-α levels induce nuclear translocation of NF-kB, leading to increased expression of miR-130a. Therefore, the association observed in our study may be explained by the interaction between miR-130a and NF-kB pathway. No direct association between increased expression of angiomiRs miR-126 and miR-328 in non-GCB cases was found. We also did not find a good hypothesis for correlation between miR-296 or miR-20b and age at diagnosis.

There was a statistically significant difference between MVD and relative expression of angiomiRs miR-16, miR-221 and miR-328, with higher levels of these antiangiomiRs in situations of lower MVD. The same was confirmed for the three antiangiomiRs and the stromal signature, i.e., higher levels of antiangiomiRs in stromal-1 subtype. Therefore, technically, the two methods had confirmed results with similar meanings.

Analyzing overall survival according to the categories of each of the clinical or laboratory variables, we observed worse outcome in groups classified as Ann Arbor stage III/IV, high IPI and CD34 quartiles III/IV. IPI and CD34 confirmed independent impact on survival of the study group. Patients with high-risk IPI showed chance of evolving to death 3.4 times higher than the rest of the group, while patients with higher MVD showed chance of evolving to death 2.0 times higher than the rest of the group. Although all microRNAs evaluated here showed no prognostic significance, we can attribute this result in part to the fact of not having homogeneously treated patients (most of them did not receive R-CHOP). We still had a large percentage of patients discharged after five years of follow-up, which may have contributed to the median survival of the group had not been reached.

Because the proangiomiRs miR-17, miR-210 and miR-296 showed increased expression in 57%, 58% and 56% of the tumor samples, respectively, and the antiangiomiR miR-20b showed decreased expression in more than 50% of the tumor samples, these could be our target candidates for therapy. Using MiRTarBase (http://mirtabase.mbc.nctu.edu.tw/php/search.php) we found the main targets related to the angiomiRs of interest detected in our study. From there, through STRING Protein Network Tool (http://string-db.org), we found the relationship of these angiomiRs targets involved in the same pathway. One example is the VEGF-A, HGS, CDKN1A and PTEN proteins. We observed that antagomiR miR-20b is involved in the regulation of genes belonging to the four exemplified networks. This demonstrates that the action of a miRNA in different pathways controls the normal functions of cells, which can simultaneously be deregulated in cancer.

Lei *et al.* [22] showed that miR-20b has an important biological role in tumor cells, promoting growth and survival of the tumor by controlling the oxygen supply. This mechanism occurs by decreasing the expression of HIF-1alpha and VEGF-A. Li *et al.* [23] studied patients with metastatic breast cancer and observed that VEGF-A and HIF-1alpha showed a higher level of expression in the group with high invasion, with negative correlation with miR-20b. Given the paucity of data in the literature regarding the function of miR-20b in cancer, it seems to be an interesting potential target in DLBCL. In addition, low miR-20b expression was related to low MVD and stromal-1 signature, although no correlation of this antiangiomiR with prognosis was observed.

High expression of miR-17 was found in several types of cancer, such as colorectal cancer, gastric cancer, hepatocellular carcinoma, glioma, basal cell carcinoma and pancreatic cancer. Zeng *et al.* [24] demonstrated that miR-17 was overexpressed in serum of patients with nasopharyngeal carcinoma. Wu *et al.* [25] showed that miR-17-5p acts as a proliferative factor for gastric cancer by inhibiting SOCS6 target. Qi *et al.* [26] evaluated the serum of patients with non-small cell lung cancer in initial stage and healthy donors and found that miR-17 showed higher expression levels in patients in early stage. Therefore, it may be used as a biomarker in the diagnosis of early stage non-small cell lung cancer. Fang *et al.* [27] observed that miR-17-5p had significantly higher levels in patients with colorectal cancer in advanced clinical stages with metastasis. They also found that PTEN is the target of miR-17-5p in colon cancer cells and their interaction is responsible for multidrug resistance. Gao *et al.* [28] also showed correlation between miR-17 and PTEN in osteosarcoma and contribution of miR-17 in the progression and metastasis of this cancer type.

Zhan *et al.* [29] studied the serum levels of miR-210 in patients with hepatocellular carcinoma who underwent arterial chemoembolization. They observed an increase in serum in the group with hepatocellular carcinoma, as well as a relationship between worse overall survival and increased serum levels after four weeks of treatment in refractory cases. Qu *et al.* [30] demonstrated that miR-210 has upregulation in colorectal cancer, with higher expression levels correlating with larger tumor size, lymph node metastasis, advanced clinical stages and worse prognosis. Wang *et al.* [31] conducted a systematic review and meta-analysis to evaluate the role of the increase in miR-210 expression in the prognosis of some cancers (breast cancer, squamous cell carcinoma of head and neck, renal cancer, sarcoma soft tissue, pediatric osteosarcoma, bladder cancer and glioblastoma). They observed that the increased expression of miR-210 has a negative impact on disease-free survival, progression-free survival and relapse-free survival.

Conflicting results are reported regarding the role of miR-296 in carcinogenesis. Wei *et al.* [32] demonstrated that miR-296 regulates the HMGA1 expression in patients with prostate cancer, noting that high levels of HMGA1 were associated with low expression levels of miR-296 and strongly associated with more advanced disease stage. The increase in miR-296 expression significantly reduced cell proliferation and prostate cancer invasion. Lee *et al.* [33] also studied the miR-296 in prostate cancer, but having Pin1 as target; increased Pin1 expression is related to tumor development and poor prognosis in prostate cancer. They demonstrated that miR-296-5p has a tumor suppressor role, with potential prognostic effect. Hong *et al.* [34] investigated the expression of miR-296 in esophageal squamous cell carcinoma and observed increased expression in affected tissues with esophagitis, esophageal carcinoma in situ and esophageal squamous cell carcinoma. They demonstrated that the reduction of miR-296 expression rendered increased survival, even in patients with metastatic lymph node disease.

In the present work we identified potential angiomiRs candidates for targeted therapy. Depending on the function of the angiomiR, two antitumor therapeutic approaches may be used: antagomirs or microRNAs that mimic the lost function (mimics) [35–37]. We looked for proangiomiRs which had increased expression (above 50%) (miR-17, miR-210 and miR-296), candidates to the antagomiR approach, trying to interrupt the angiogenesis process by inhibiting miRNA expression. Regarding to antiangiomiRs, miRNA mimics may be used to increase the expression of the miRNA of interest. MiR-20b was the only one to show decreased expression in more than 50% of DLBCL patients and it is likely an important target for clinical evaluation.

## MATERIALS AND METHODS

### Patients

We identified 124 patients treated at Sao Paulo Hospital between 2000 and 2010 whose paraffin blocks were available for immunohistochemical and molecular analysis. All 84 eligible cases were confirmed as DLBCL. We excluded nine HIV-positive cases, five T-cell/histiocyte DLBCL, 12 primary central nervous system and 14 cases that could not be “de novo” DLBCL. Demographic and clinical data, such as age, sex, histological diagnosis, Ann Arbor clinical stage and International Prognostic Index (IPI) were obtained from our databank and patients' charts. This study was approved by Ethics Committee of Sao Paulo Hospital/UNIFESP (CEP 0002/11). Due to the retrospective characteristic of the study, written consent was not required.

### Tissue microarray (TMA)

We constructed a tissue microarray (TMA) using paraffin blocks and Beecher Instruments equipment (Estigen, Tartu, Estonia). All H&E-stained DLBCL slides demonstrated more than 70% of tumor area, with no significant tissue necrosis. The samples were represented in duplicate in the receptor TMA block.

### Immunohistochemical analysis (Table 1)

**Table 1:** Specifications of primary antibodies used in the TMA

Marker	Clone	Brand	Dilution	pH antigen retrieval	Time of antigen retrieval	Positive Control
**CD10**	56C6 mouse	Dako	1:2	Tris-EDTA pH 9.0	20 min 97°C	Palatine tonsil
**BCL6**	LN22 mouse	Novocastra	1:100	Citrate pH 6.1	20 min 97°C	Palatine tonsil
**MUM1**	MUM1p mouse	Novocastra	1:1200	Citrate pH 6.1	20 min 97°C	Palatine tonsil
**CD68**	KP1 mouse	Dako	1:6000	Citrate pH 6.1	20 min 97°C	Palatine tonsil
**SPARC**	H-90	Santa Cruz Biotechnology	1:500	Citrate pH 6.1	20 min 97°C	Kidney
**CD34**	QBEnd10 mouse	Dako	1:800	Citrate pH 6.1	20 min 97°C	Prostate

**Table 2:** Clinical characteristics of patients, according to the classification in the molecular subtypes and cellular origin [13]

	GCB	ABC	P
**Gender**			
Female	19 (48.7%)	17 (54.8%)	0.6108
Male	20 (51.3%)	14 (45.2%)	
**Age**			
<60 years	17 (43.6%)	15 (48.4%)	0.6890
≥ 60 years	22 (56.4%)	16 (51.6%)	
**Ann Arbor Staging**			
I/II	17 (43.6%)	12 (38.7%)	0.6805
III/IV	22 (56.4%)	19 (61.3%)	
**IPI**			
0-1 (low)	11 (28.2%)	5 (16.1%)	0.3994
2-3 (intermediate)	18 (46.1%)	19 (61.3%)	
4-5 (high)	9 (23.1%)	7 (22.6%)	
Not assessed	1 (2.6%)	0	

**Table 3:** Overall survival analysis of patients included in the study

Variable	N (%)	Median Overall Survival (months)	p (Log-Rank)
**Ann Arbor Staging**			
I/II	33 (39.3%)	Not reached	**0.0256**
III/IV	51 (60.7%)	24.27	
**IPI**			
0-1 (low)	17 (20.5%)	Not reached	**0.0236**
2-3 (intermediate)	46 (55.4%)	Not reached	
4-5 (high)	20 (24.1%)	8.47	
**Hans**			
GCB	39 (55.7%)	Not reached	0.1472
ABC	31 (44.3%)	21.6	
**CD68**			
Low expression	20 (24,4%)	Not reached	0.5965
High expression	62 (75.6%)	74.67	
**SPARC**			
Positive	32 (47.1%)	Not reached	0.9378
Negative	36 (52.9%)	Not reached	
**MVD — automated counting**			
Quartiles I/II	42 (50%)	Not reached	**0.0363**
Quartiles III/IV	42 (50%)	23.9	
**MVD — manual counting**			
Quartiles I/II	40 (50%)	Not reached	0.4041
Quartiles III/IV	40 (50%)	Not reached	
**Stromal Signatures**			
Stromal-1	32 (44.4%)	Not reached	0.6157
Stromal-2	40 (55.6%)	Not reached	

The variables include: Ann Arbor staging I/II *vs* III/IV, IPI low *vs* intermediate *vs* high, molecular subtypes-GCB *vs* non-GCB or ABC, expression level of immunohistochemical markers CD68, SPARC, CD34 and stromal signatures

CD10, Bcl-6 and MUM-1 were used to classify cases according to their cellular origin [13]: 0 (0-10% positive cells); 1 (10-25%); 2 (25-50%); 3 (≥ 50%) (three slides of the TMA block for each marker were analyzed in duplicate). Expression of CD68 (same criteria above) and SPARC (< 5% negative and ≥ 5% positive) were used as stromal-1 markers [18]. To analyze the expression of CD34 (MVD, stromal-2), we performed automated and manual microvessel counting across the field of TMA. For automated analyses, we considered the number of CD34+ objects/100μm^2^ of TMA area. [Formula = (Microvessel Density - number of vessels per unit area (μm^2^) × Total Stain Area (μm^2^)/100], excluding areas inferior to 20μm^2^ [10]. In parallel, we conducted a manual counting of microvessels in the fullfield of TMA at 400X magnification. Those which appeared continuous microvessels were considered only once. Slide images were captured using ScanScope AT Turbo equipment (Aperio Technologies, Vista, CA, USA). Two independent observers classified slides using a semi-quantitative method (N.M.B. and A.C.A.).

### microRNA extraction and qPCR reaction in paraffin embedded samples

*miRNA extraction was performed using paraffin RecoverAll™ Total Nucleic Acid Isolation Kit for FFPE Tissues (Applied Biosystems, Foster City, CA). The quantitative real*-time PCR was performed using TaqMan Assays Small RNA kit (Applied Biosystems, Foster City, CA). ProangiomiRs Let-7f (*Assay ID* 000382), miR-17 (*Assay ID* 002308), miR-18a (*Assay ID* 002422), miR-19b *(Assay ID* 002425), miR-126 (*Assay ID* 002228), miR-130a (*Assay ID* 000454), miR-210 (*Assay ID* 000512), miR-296 (*Assay ID* 000527) and miR-378 (*Assay ID* 002243) and antiangiomiRs miR-16 (*Assay ID* 000391), miR-20b (*Assay ID* 001014), miR-92a (*Assay ID* 000431), miR-221 (*Assay ID* 000524) and miR-328 (*Assay ID* 000543) were selected for analysis. U18 (*Assay ID* 001204) and RNU44 (*Assay ID* 001094) were used to normalize reactions. Nine palatine tonsils samples served as negative controls. MicroRNAs were considered differentially expressed when levels of tumor samples were 1.2-fold higher or lower than control samples using 2^−ΔΔCt^ formula [38].

### microRNA extraction and qPCR reaction in serum samples

We extracted total RNA from 200μL of serum (in duplicates) of 10 control samples from normal healthy individuals, 39 samples from patients with DLBCL at diagnosis and 19 samples from patients with DLBCL at the time of relapse, using miRNeasy Serum/plasma Kit (Qiagen, Dossseldorf, Germany). We determined the concentration of the extracted total RNA from serum samples using the DeNovix DS-11 spectrophotometer. 10ng of total RNA was used for cDNA synthesis with TaqMan Assays Small RNA kit (Applied Biosystems, Foster City, CA). The expression of RNU44 was used as calibrator for the qPCR, using 7500 Real Time System (Applied Biosystems, Foster City, CA). Samples with CT value greater than 38 were considered negative, according to Song *et al*. [39].

### Statistical analysis

The associations between the variables of interest were tested with the chi-square test (or Fisher's exact test). To estimate the significance of the difference between the mean or median we performed the t test (unpaired or Mann-Whitney, respectively) or the analysis of variance (OneWay ANOVA with Post Hoc Test or OneWay ANOVA with Tukeys's Multiple Comparison test). The Pearson correlation was used to analyze possible continuous numerical associations. Overall survival was calculated as the time between start of treatment and death related to the disease. Deaths not related to the disease or dropouts were censored. The overall survival curves were made according to the Kaplan-Meier method and compared using the log-rank test. For all statistical tests, the significance level of 5% (α=5%) was used. Statistical analysis was performed with SPSS version 8.0 for Windows. The gene expression graphs were constructed with the GraphPad Prism version 5.0 (www.graphpad.com).

## SUPPLEMENTARY FIGURES


