# Supplementary material for: Angiomirs expression profiling in diffuse large B-Cell lymphoma
Source: Oncotarget. 2015 Dec 15;7(4):4806–16. doi: 10.18632/oncotarget.6624 (PMC4826244; doi:10.18632/oncotarget.6624)
Supplement: Supplementary file 1 [file oncotarget-07-4806-s001.pdf]

SUPPLEMENTARY FIGURES

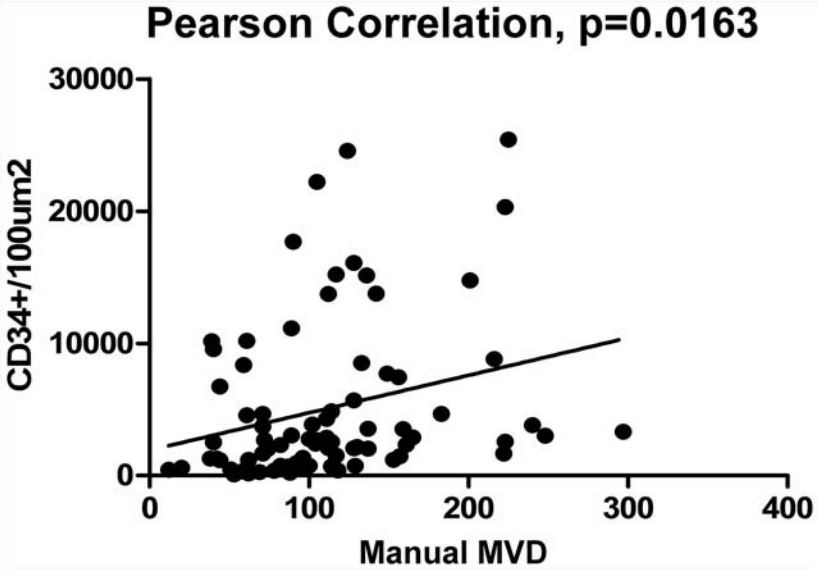

Supplementary Figure S1: Pearson correlation between manual and automated evaluation of MVD (CD34+/100 µm^2).

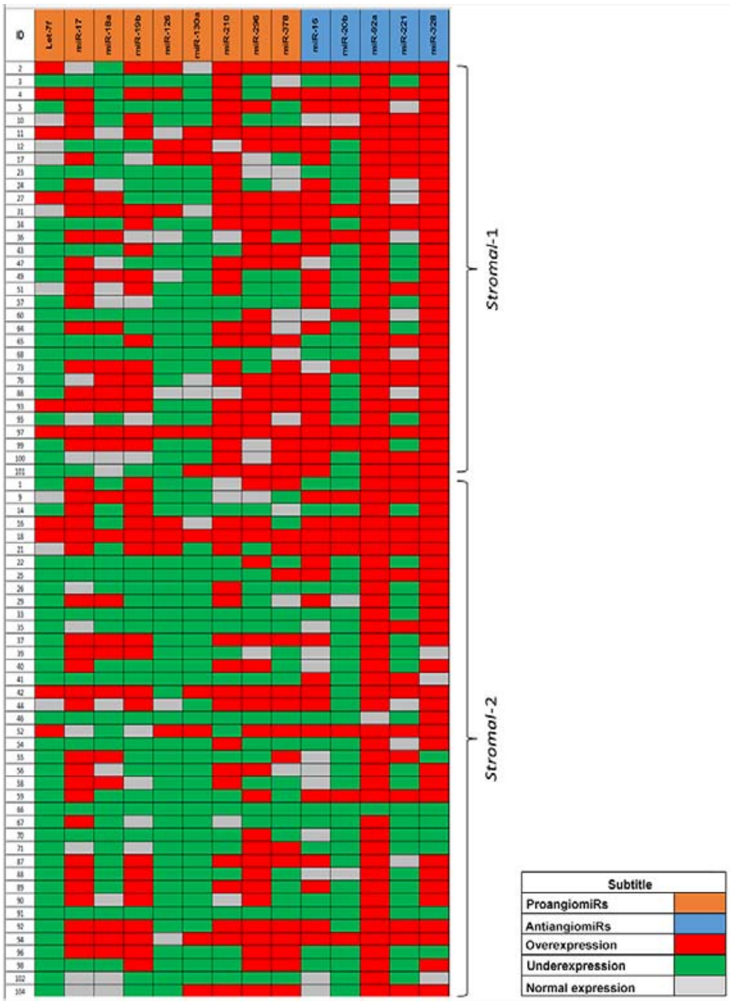

Supplementary Figure S2: Expression of angiomiRs in cases classified as stromal-1 and stromal-2.

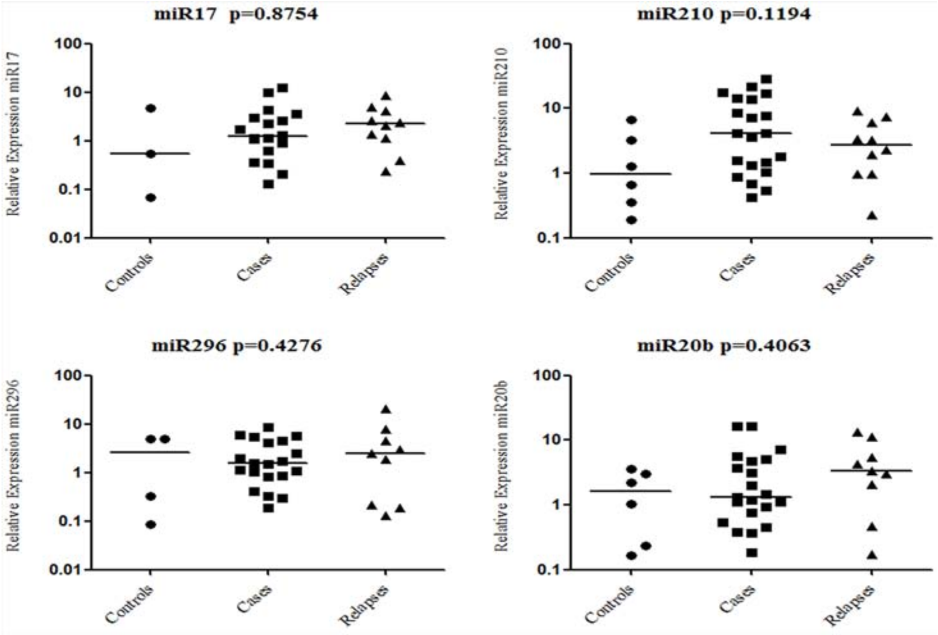

Supplementary Figure S3: Expression of angiomiRs in serum samples.
